# Multifunctional Cyclic Olefin Copolymer-Titanium Dioxide-Based Nanocomposites for Enhanced Antibacterial and Biocompatible Properties

**DOI:** 10.1021/acsomega.4c10536

**Published:** 2025-04-08

**Authors:** Zakia Riaz, Murad Ali, Amna Didar Abbasi, Kamran A. Khan, Omer Aydin

**Affiliations:** † Department of Aerospace Engineering, Khalifa University of Science and Technology, Abu Dhabi 127788, United Arab Emirates; ‡ NanoThera Lab, Drug Application and Research Center (ERFARMA), Erciyes University, Kayseri 38039, Turkey; § Department of Mechanical & Nuclear Engineering, Khalifa University of Science and Technology, Abu Dhabi 127788, United Arab Emirates; ∥ Advanced Digital & Additive Manufacturing (ADAM) Group, Khalifa University of Science and Technology, Abu Dhabi 127788, United Arab Emirates; ⊥ School of Chemical and Materials Engineering, National University of Sciences and Technology, Islamabad 44000, Pakistan; # Department of Biomedical Engineering, Erciyes University, Kayseri 38039, Turkey; ¶ Clinical Engineering Research and Implementation Center (ERKAM), Erciyes University, Kayseri 38030, Turkey; ∇ Nanotechnology Research and Application Center (ERNAM), Erciyes University, Kayseri 38039, Turkey

## Abstract

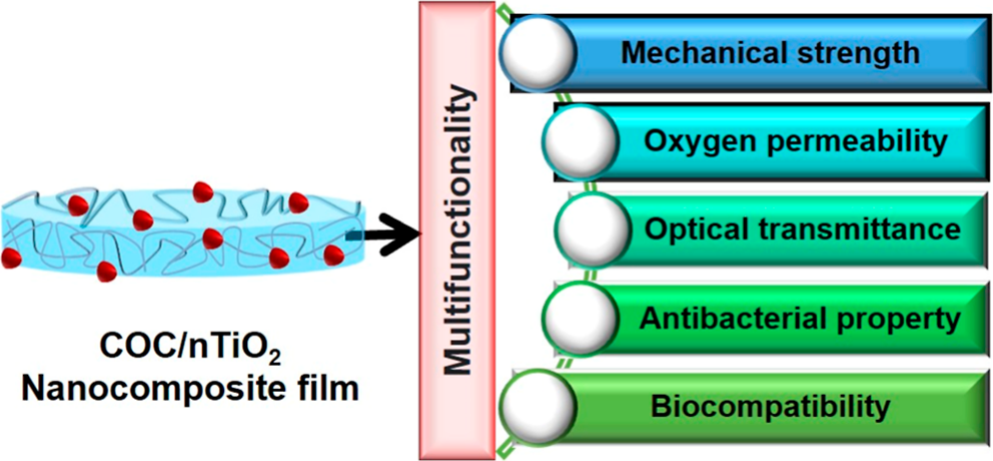

The growing demand for multifunctional materials in biomedical and packaging applications necessitates the development of advanced nanocomposites with superior mechanical, barrier, and antibacterial properties. This study presents cyclic olefin copolymer (COC)/titanium dioxide (TiO_2_) nanocomposite films fabricated through the facile solution casting method, incorporating TiO_2_ at concentrations of 1, 3, and 5 wt %. Structural and morphological analyses confirmed effective TiO_2_ dispersion at lower concentrations, while agglomeration was observed at 5 wt %. The incorporation of TiO_2_ significantly enhanced barrier properties, reducing water vapor permeability from 80.67 to 29.99 g/m^2^ h and oxygen permeability from 7.66 to 2.78 mg/mL. Mechanical properties showed marked improvement, with tensile strength increasing by 113% and tensile modulus by 81.3% at 3 wt % TiO_2_. Antibacterial tests demonstrated efficacy against *Escherichia coli* and *Staphylococcus aureus*, while cytotoxicity studies confirmed the biocompatibility of the films. These findings highlight the potential of COC/TiO_2_ nanocomposites for applications, such as antibacterial coatings, wound healing patches, and high-performance packaging.

## 1 Introduction

Over the past years, remarkable progress has been made in the development of new polymers that can be employed as potential materials of choice in almost every field of life.^1^ However, pure polymer alone does not have all of the required physical, chemical, and biological properties. To improve these shortcomings and obtain a synergistic set of properties, inorganic–organic hybridizations have been developed.^2,3^ The incorporation of various inorganic nanofillers, particularly metal oxide nanoparticles (such as zinc oxide, iron oxide, silicon dioxide, and titanium dioxide), is significant because these nanoparticles improve various properties of polymers like thermal, physiochemical, mechanical, antimicrobial, and barrier properties.^4−6^ The development of high-performance and eco-friendly nanocomposite materials based on polymer matrices having different sizes of metal oxide nanoparticles has attracted much attention in the past decade given the need to create new materials having numerous applications in food and packaging industries.^5,7,8^

Among various metal oxide nanoparticles, titanium dioxide nanoparticles (nano-TiO_2_) exhibit promising applications due to their low cost, nontoxicity, inertness, photocatalytic antibacterial properties, and biocompatibility.^9^ United States Food and Drug Administration also approved its uses as human drug and food contact material.^10−13^ It has been reported that nano-TiO_2_ tends to agglomerate which causes reduction in their efficiency because of decrease in the surface-to-volume ratio.^14^ However, Al-Sagheer and Merchant and Qian et al. demonstrated that blending nanosized TiO_2_ and organic polymers results in the reduction of their spontaneous agglomeration inside polymer matrix that directly affects the properties of the resultant nanocomposite films.^15,16^ These studies revealed that a combination of nano-TiO_2_ with organic polymers improves the barrier, mechanical, thermal, and antibacterial properties of the resultant nanocomposite system.^17^ Packaging systems consisting of nano-TiO_2_-based composite materials receive general attention because nano-TiO_2_ exhibits superior photocatalytic antibacterial properties by the production of free radicals like singlet oxygen and peroxides that interact with bacterial cells and kill them.^4,9,18^ Li et al.^19^ synthesized a novel packaging material by adding nanopowder (anatase TiO_2_, rutile TiO_2_, Ag, and kaolin) in polyethylene film to preserve Chinese jujube along with improving its shelf life. Similarly, Haldorai and Shim^20^ examined the antibacterial and photocatalytic activities of TiO_2_-encapsulated chitosan-based nanohybrid films.

Several biocompatible polymers have been recently discovered that incorporate metal oxide nanoparticles and enhance the functional properties of resultant nanocomposite films.^4,7,8,18^ Cyclic olefin copolymer (COC), also commercially known as Topas, is an emerging thermoplastic polymer formed by the copolymerization of ethylene and norbornene functionalities.^21^ COC polymer has numerous applications in optics, electronics, biomedical, and packaging areas. It has excellent mechanical and barrier properties, exhibiting resistance toward various chemicals as well as bacteriostatic properties to most common bacterial strains.^22−25^ Few studies have been conducted in the past related to polymeric blends and hybrid nanocomposite films of the COC polymer. Riaz et al.^21^ studied the effect of the addition of PLLA polymers on the mechanical and thermal properties of COC polymer film. Ain et al.^26^ prepared a biocompatible hybrid nanocomposite system for bone grafting applications by combining COC polymer with varying concentrations of hydroxyapatite (HA) nanoparticles. Saleem et al.^24^ prepared COC/few layer graphene-based nanocomposite film systems that showed remarkable antimicrobial and mechanical properties and can be used as an active packaging material.

Previous studies on TiO_2_-based polymer nanocomposite films face challenges like nanoparticle agglomeration and reducing optical and mechanical properties.^27^ Limited biocompatibility assessments restrict their biomedical applications.^28^ Many studies focus on isolated properties, such as ionic conductivity and tensile strength (TS), neglecting multifunctionality. Moreover, complex methods like sol–gel also hinder scalability for practical applications.^29−31^ These issues must be addressed to unlock the full potential of the TiO_2_-based nanocomposite films. Taking into consideration these shortcomings, herein, an effort has been made to develop multifunctional COC polymer-based nanocomposite material for various applications in the biomedical field. The varying content of TiO_2_ nanoparticles (1, 3, and 5 wt %) was blended with the COC polymer through a simple solution casting method, while pure COC film was used as a positive control. The prepared nanocomposite films were subjected to various characterizations such as optical, morphological, antibacterial, and mechanical. It was observed that nanocomposite material with varying TiO_2_ content displayed multifunctional characteristics, such as improved mechanical, barrier, optical, and antibacterial properties. It was worth mentioning that the nanocomposite system exhibited cytocompatibility toward L929 cell lines. These experimental outcomes suggest that this nanocomposite system with high-performance multifunctional characteristics might be useful as packaging material, in wound healing antibacterial patches, and in tissue engineering field.

## 2 Experimental Section

### 2.1 Materials

COC also known as Topas (Topas-8007) was obtained from TICONA GmbH (Germany), and chloroform was purchased from Sigma-Aldrich (Burlington, USA). For the synthesis of nano-TiO_2_, titanium isopropoxide (<97%), 2- methoxyethanol (99%), and ethanolamine (99%) were obtained from Sigma-Aldrich. All chemicals used were of analytical grade.

### 2.2 Synthesis of Nano-TiO_2_

The sol–gel method was used to synthesize titanium dioxide nanoparticles.^32^ In this method, titanium(IV) isopropoxide, ethanolamine, and ethylene glycol monomethyl ether (1:0.5:4) were mixed in a round-bottom flask, stirred, and refluxed for 1 h under an inert environment at room temperature. Afterward, the temperature increased to 80 °C with continued stirring for 1 h. The temperature was further increased to 120 °C with continuous stirring for an additional 2 h under an inert environment. A yellowish-orange color appeared, which confirmed the successful formation of TiO_2_ sol. At last, to obtain TiO_2_ nanoparticles, this sol was calcined for 6 h at 500 °C.

### 2.3 Preparation of COC/Nano-TiO_2_ Nanocomposite Films

The solution casting method was used for the preparation of nanocomposite films. Before preparation, both COC polymer and nano-TiO_2_ powder were kept in the oven for about 5 h for reduction of moisture content. COC polymer and nano-TiO_2_ powder were dissolved separately in dry chloroform at room temperature. The nano-TiO_2_ solution was sonicated for 1 h before mixing with the COC polymer solution. The resultant mixture solution was stirred for almost 10 h at room temperature as well as sonicated for 2 h in an ultrasonic bath sonicator. Afterward, the solution was poured into a glass Petri dish and left overnight for complete evaporation of solvent at room temperature, followed by drying in a vacuum oven at 50 °C for about 5–6 h to remove any leftover traces of solvent. For the preparation of COC/nano-TiO_2_ nanocomposite films, the concentration of nano-TiO_2_ was kept at 1, 3, and 5 wt % in a fixed amount of COC polymer (0.5 g). These prepared films were cut into samples of different dimensions according to the requirements of characterization techniques.

TiO_2_ concentrations of 1, 3, and 5 wt % are selected as they produce high-quality films with uniform dispersion and enhanced properties, avoiding agglomeration at higher concentrations and insufficient enhancement at lower levels. The solution casting method is chosen for its simplicity, cost-effectiveness, and ability to ensure excellent nanoparticle dispersion, and uniformity.

### 2.4 Structural and Morphological Characterizations

#### 2.4.1 X-ray Diffraction

X-ray diffractograms of pure COC polymer, nano-TiO_2_, and nanocomposite films were obtained by using an Xpert’s PRO PAN analytical X-ray diffractometer with a CuK_α_ radiations (λ = 1.5405 Å) using operational current and voltage of 40 mA and 40 kV at room temperature with a scanning rate of 2° per minute in the range of 2θ = 10–70°.

#### 2.4.2 Fourier Transform Infrared Spectroscopy

Fourier transform infrared (FTIR) analysis was carried out to study possible interactions among functional groups of the COC polymer and nano-TiO_2_. FTIR spectra were recorded by using (ATR–FTIR JASCO7890) at a resolution of 1 cm^–1^ on the reflection mode from 4000 to 500 cm^–1^.

#### 2.4.3 Scanning Electron Microscopy and Atomic Force Microscopy

Morphological and compositional analyses of nanocomposite films were carried out by using scanning electron microscopy (SEM) (JEOL-instrument JSM-6490A. Before using SEM (JEOL JFC-1500), an ion sputtering machine was used for gold sputtering (∼15 nm) of all nanocomposite films, and then, samples were mounted on aluminum stubs for examination. The prepared nanocomposite films were cryofractured after immersing them in liquid nitrogen to observe the cross-sectional area. The aforementioned SEM equipped with an energy-dispersive X-ray spectroscopy (EDX) detector was also utilized for compositional and distribution analysis of prepared nanocomposite films. Surface roughness measurements were carried out using atomic force microscopy (AFM) (Digital instrument nasoscope IIIa System).

### 2.5 Water Vapor Permeability

Barrier properties of nanocomposite films were determined by studying water vapor transmission rate per unit area (WVTR) at room temperature using ASTM E96–95 as the standard method. Samples were wrapped around the top of 10 mL of distilled water-containing glass cups with the help of Teflon tape to avoid airflow. The initial weight of the cups was recorded and then placed in an oven at 40 °C for 24 h. The glass cups with samples were weighed again to calculate water vapor permeability (WVP) values. At least five samples of each nanocomposite film with a specific concentration were tested to obtain the average WVP values.

### 2.6 Oxygen Permeability

The Winkler method was used to study oxygen permeability (OP) values of COC/nano-TiO_2_ nanocomposite films.^33^ The mouths of reagent bottles with distilled water were covered with nanocomposite films by using Teflon tape and were stored for 24 h. To estimate OP values, one bottle was kept closed, and the other was kept open for positive and negative controls. OP values were calculated in mg/mL, and at least five replicates of each nanocomposite film were tested.

### 2.7 Optical Transmittance

Optical transmittance analysis of nanocomposite film was performed by following the technique from the literature.^34^ An UV–vis spectrophotometer (PerkinElmer, LAMBDA 35 UV/vis spectrophotometer) was used, and analysis was conducted in the visible region (400–800 nm). Samples were cut down in rectangular shape, and five replicates of each nanocomposite film were used to get the average transmittance value.

### 2.8 Mechanical Testing

Mechanical properties of nanocomposite films were studied at room temperature with the help of a Trapezium-X Universal Testing Machine (AG-20KNXD Plus) assembled by Shimadzu Corporation. Mechanical testing was performed at a cross-head speed of 5 mm/min where samples were cut into dimensions of 10 × 90 mm (width × length) having a gauge length of 20 mm. The tensile experiment was carried out by taking five samples of each nanocomposite film to obtain the average values.

### 2.9 Antibacterial Study

The antibacterial properties of nanocomposite films were tested against two bacterial strains, *Escherichia coli* and *Staphylococcus aureus* by using the disc diffusion method. The bacterial strains were grown in freshly prepared nutrient broth media and incubated at 37 °C for 24 h. These bacterial strains were added by putting a 100 μL bacterial culture suspension in each sterilized Petri dish. Later, equal-sized discs of nanocomposite films were placed on these bacterial lawns. Ciprofloxacin was used as a positive control, and pure polyether sulfone film was used as a negative control. Subsequently, these Petri dishes were incubated at 37 °C for 24 h. The antibacterial activity was determined by measuring the diameter of the zone of inhibition formed by nanocomposite films. All samples were tested in triplicates. The time-dependent antibacterial experiment was carried out by carefully forming nanocomposite films with varying TiO_2_ contents on the bottom of 24-well plates. Afterward, 1 mL of bacterial solution was poured on them and left on a mechanical shaker for different time intervals of 4, 8, 12, and 24 h at a speed of 90 rpm and a temperature of 37 °C. At specific time intervals, 100 μL of the bacterial solution is transferred to another 96-well plate, and by using a Synergy H1 multimode microplate reader (BioTek), and optical density (OD) values were calculated to determine the antibacterial activities of these composite films. The bacterial cell morphology before and after treatment with a nanocomposite film was evaluated using SEM. Initially, a bacterial solution was dropped on silicon wafers (a few drops of 2% glutaraldehyde solution were added) and was allowed to dry at room temperature for almost 3 h. After the fixation process, the dehydration of bacterial cells was carried out using a series of ethanol solutions (50%, 70%, 90%, and 100%). Afterward, these silicon wafers were dried with liquid nitrogen to preserve the morphology of the bacterial cells. Finally, gold sputtering is performed before analysis.

### 2.10 Cytocompatibility Study

Cell cytotoxicity studies of pure COC film and nanocomposite films were carried out by using L929 cells. The L929 cells were bought from the American Type Culture Collection (ATCC Rockville, MD, USA). These cells were cultured in a medium (αMEM) containing 1% antibiotics (penicillin–streptomycin) with 10% FBS at 37 °C. Pure COC and nanocomposite films were prepared carefully at the bottom of the 24-well plates. Later, a 100 μL solution of cells with a cell density of 4000 cells/100 mL was transferred to each well. These plates were then incubated at 37 °C for 24 and 48 h, respectively. After the required time period, the cell viability analysis was carried out by employing a cell counting kit 8 assay (CCK-8 water-soluble tetrazolium salt). For cell viability measurements, 10 μL of CCK-8 was transferred into 24-well plates having cells with pure COC and nanocomposite films, and these plates were again incubated at 37 °C for 2 more hours. Lastly, with the help of a flash spectral scanning multimode reader, OD values were determined by measuring the absorbance at 450 nm. All experiments were performed in triplicates.

#### 2.10.1 Live/Dead Fluorescence Assay

For live/dead fluorescence assay, L929 cells were first washed with PBS solution (pH = 7.4) after the specific incubation period (24 and 48 h). The cells were cultured in a staining mixture composed of 2 μM Calcein-AM, 4 μM propidium iodide (PI), and PBS solution for approximately 30–40 min. Confocal laser scanning microscopy was used to analyze the results. The living cells having Calcein-AM displayed a green fluorescence, while the dead cells with PI exhibited a red fluorescence.

## 3 Results and Discussion

### 3.1 Structural and Morphological Analysis

**Figure 1:**
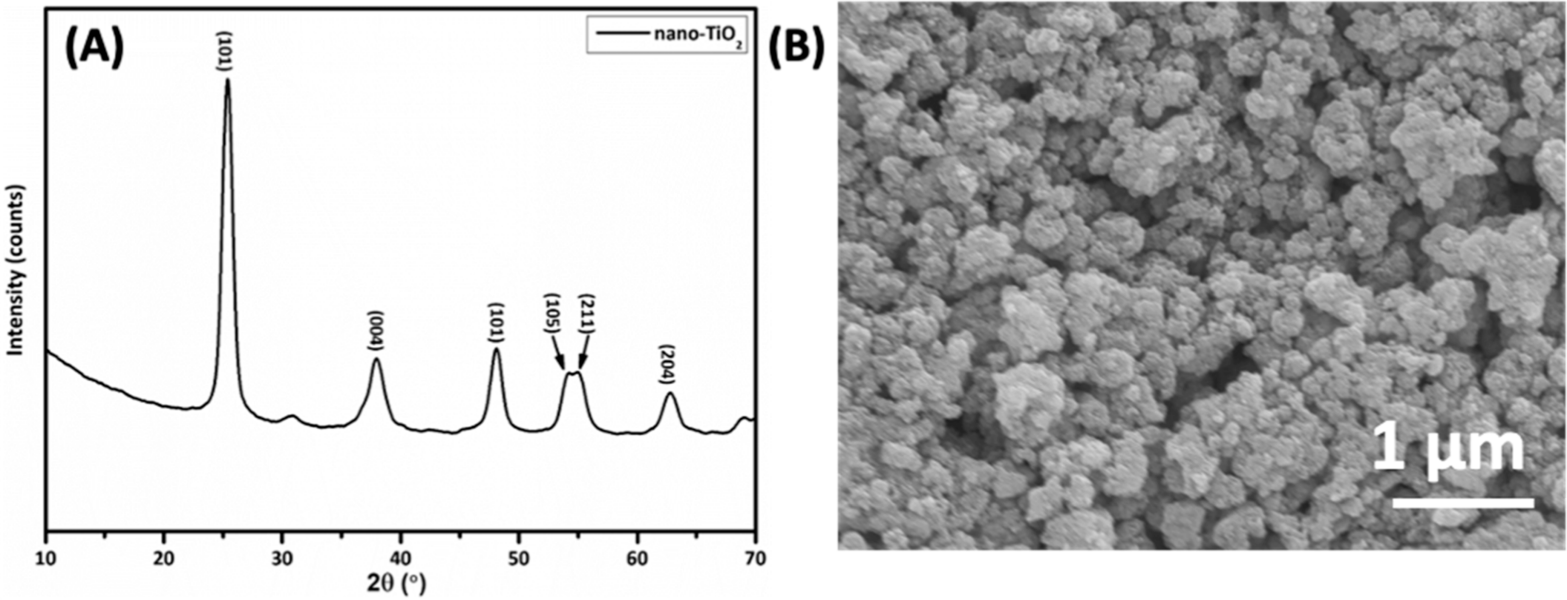
(A) XRD image of synthesized nano-TiO_2_ and (B) SEM micrograph of powder nano-TiO_2_. The scale bar is 1 μm.

**Figure 2:**
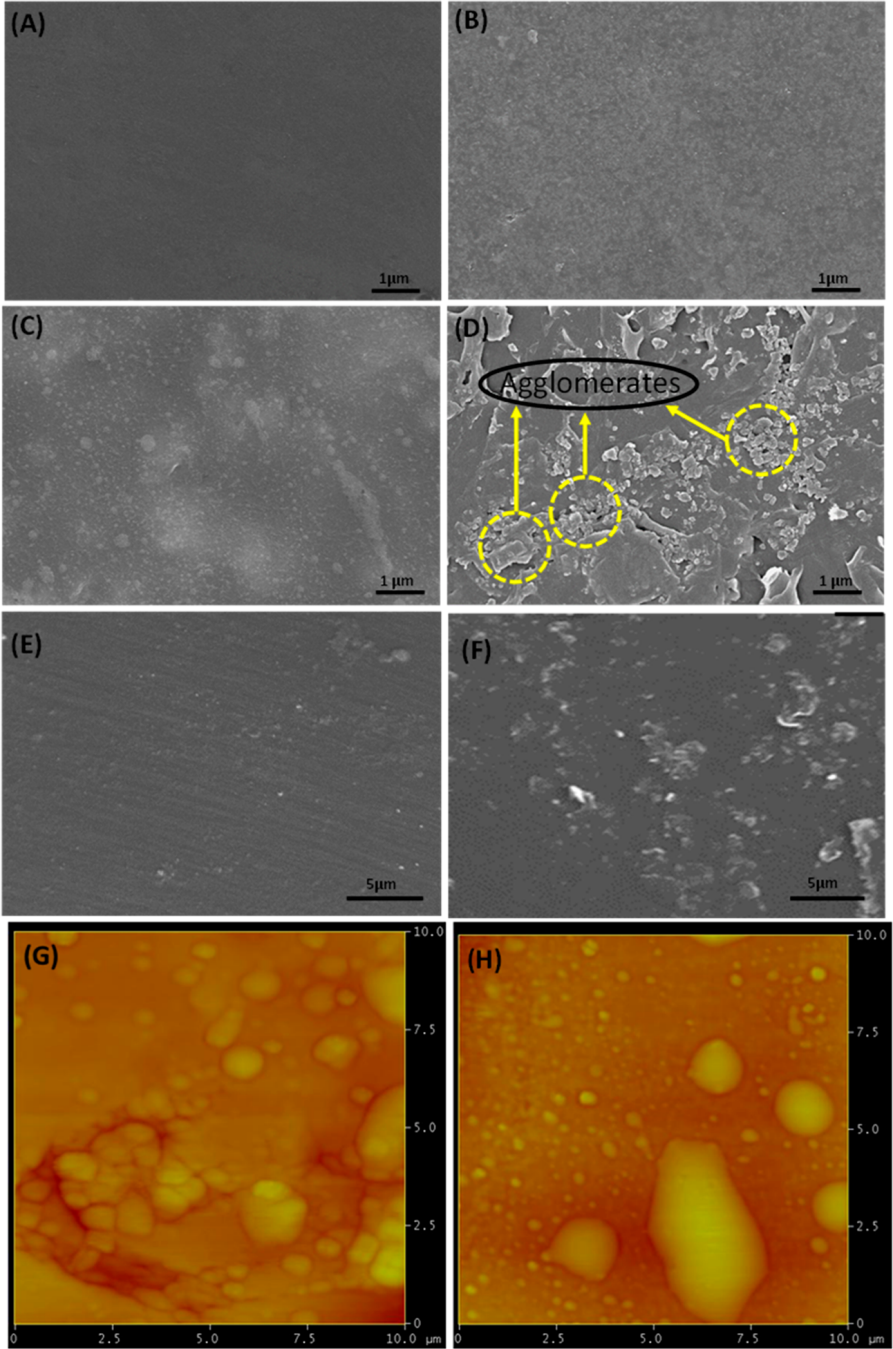
SEM images presenting surface morphology of (A) pure COC polymer film. COC polymer having nano-TiO_2_ concentration of (B) 1, (C) 3, and (D) 5 wt %. SEM micrographs presenting cross-section view of nanocomposite films with nano-TiO_2_ concentration of (E) 1 and (F) 5 wt %. AFM images of developed COC-based nanocomposite films with (G) 1 and (H) 5 wt % nano-TiO_2_ particles.

To evaluate the effect of nano-TiO_2_ addition on various properties of the COC polymer, COC/nano-TiO_2_ nanocomposite films were prepared. Initially, structural and morphological analyses of filler material (nano-TiO_2_) were carried out using X-ray diffraction (XRD) and SEM (Figure 1). Figure 1A represents the XRD pattern of synthesized nano-TiO_2_ that matches the reported diffraction pattern in the literature.^35^ Similarly, the SEM image depicts the spherical morphology of nano-TiO_2_ with size ranges between 5 and 15 nm (Figure 1B). To visualize the dispersion of nano-TiO_2_ in the COC polymer, SEM characterization was carried out. Figure 2 represents SEM images of a pure COC polymer film as well as COC/nano-TiO_2_ nanocomposite films with varying concentrations of nano-TiO_2_. The featureless and smooth surface of the COC polymer film indicates its completely amorphous nature (Figure 2A), and similar results of the amorphous nature of the COC polymer was reported by Nazir and Iqbal.^36^ Morphology of nanocomposite film having 1 wt % nano-TiO_2_ shows that nanoparticles uniformly dispersed within the polymer matrix without the formation of any visible agglomerate (Figure 2B). However, surface morphology investigation of nanocomposite films with increasing concentration of nano-TiO_2_ up to 3 wt % shows some clusters appearing inside the polymer matrix although nanoparticles are still dispersed inside the polymer matrix material (Figure 2C). When the concentration is further increased up to 5 wt %, large cluster sizes of agglomerated nano-TiO_2_ started forming throughout the polymer matrix, as shown in Figure 2D. SEM images of the cross-sectional view of nanocomposite films also revealed that agglomeration in film increases by increasing nano-TiO_2_ contents (Figure 2E,F). The dispersion of nano-TiO_2_ inside the polymer matrix is directly linked with its concentration, and it is expected to affect the other properties of nanocomposite films as discussed later.^1,37^ Ain et al.^26^ studied the dispersion of various concentrations of HA nanoparticles in the Topas polymer. At the lower concentration of HA nanoparticles (10 wt %), they were uniformly dispersed inside the Topas polymer, followed by the formation of agglomerates at higher concentrations (20 wt %). Similarly, Siripatrawan and Kaewklin investigated concentration-dependent dispersion of TiO_2_ nanoparticles in chitosan polymer, and better dispersion was achieved at <2 wt % concentration.^9^ AFM characterization revealed that the surface roughness of nanocomposite films increases by increasing the concentration of nano-TiO_2_. Figure 2G,H depicts that surface roughness of nanocomposite film increases from *R*_a_ = 26.862 to 37.238 nm by increasing nano-TiO_2_ content from 1 to 5 wt % in nanocomposite films, respectively. He et al.^37^ fabricated gelatin-TiO_2_ based nanocomposite films and reported that the surface roughness of these films was increased in the presence of TiO_2_ nanoparticles.

**Figure 3:**
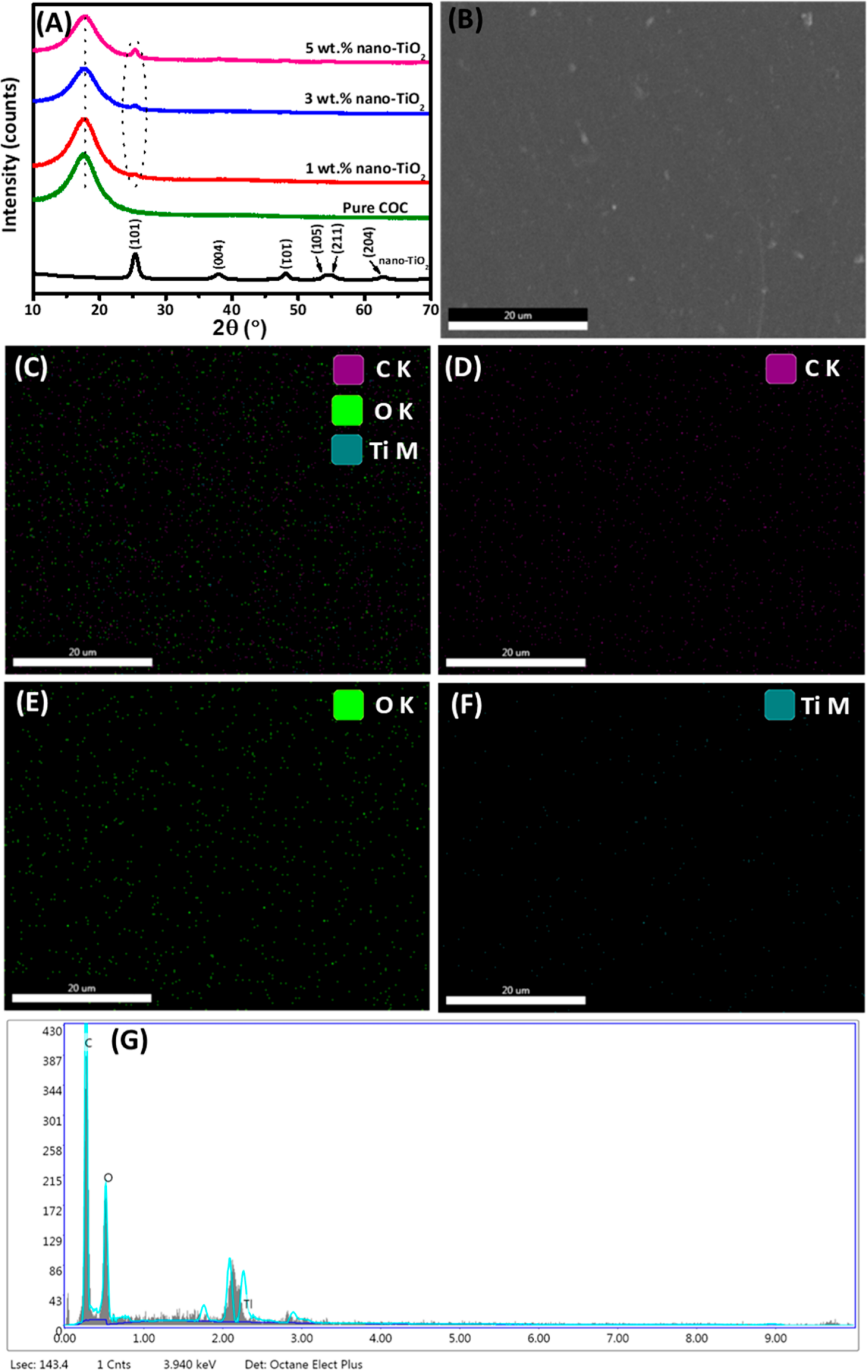
Structural and compositional characterization of developed nanocomposite films. (A) XRD diffraction patterns of nano-TiO_2_, pure COC polymer, and COC/nano-TiO_2_ nanocomposite films containing 1, 3, and 5 wt % nano-TiO_2,_ respectively. (B–F) SEM–EDX images of COC/3 wt % nano-TiO_2_ composite film and (G) EDX spectrum of COC/3 wt % nano-TiO_2_ nanocomposite film.

The developed nanocomposite films were characterized for their structural and compositional properties, as shown in Figure 3. X-ray crystallography of COC/nano-TiO_2_ nanocomposite films was studied to investigate the effect of nano-TiO_2_ addition in the COC polymer matrix (Figure 3A). X-ray diffractograms of pure COC polymer film, nano-TiO_2_, and COC/nano- TiO_2_ nanocomposites with varying concentrations of nano-TiO_2_ from 1 to 5 wt % are displayed in Figure 3A. The XRD pattern of COC polymer exhibits a broad hump that indicates its completely amorphous nature.^21^ The XRD pattern of nano-TiO_2_ represents several distinct crystalline peaks at 2θ = 25.3, 38.0, 47.8, 54.0, and 62.5° corresponding to the planes of (101), (004), (105), (211), and (204) contributes to the detection of its conventional anatase phase.^32,38^ It has been observed that the characteristic diffraction peak of nano-TiO_2_ at 2θ = 25.3°, corresponding to its anatase crystal phase (101) appears in the XRD patterns of nanocomposites at all nano-TiO_2_ concentrations. However, the intensity of this diffraction peak gradually increases with an increase in the concentration of nano-TiO_2_ from 1 to 5 wt %. The diffraction peak is much more visible in the case of 5 wt % nano-TiO_2_, the diffraction peak is much more visible. Similar findings were described by Siripatrawan and Kaewklin,^9^ where the intensity of the characteristic diffraction peak of anatase nano-TiO_2_ (2θ = 25.3°) was increased gradually with the increasing concentration from 0.25 to 1 wt % in Chitosan-nano-TiO_2_ nanocomposites. Moreover, the appearance of any new peak after film development confirms a new phase formation from the resultant mixture of matrix and reinforcement materials. However, XRD patterns revealed no new peak formation, confirming the successful development of COC/nano-TiO_2_ nanocomposite films.

The X-ray mapping and elemental compositional analyses were carried out for the developed nanocomposite films using EDX, as presented in Figure 3B–G. The X-ray mapping is utilized to examine the distribution and dispersion of nano-TiO_2_ inside the COC polymer matrix. A uniform distribution of a nanofiller in the respective matrix is always desirable to achieve the required properties. The concentration of nanofiller plays a critical role in presenting uniform distribution inside the matrix. Therefore, based on SEM characterization, where 3 wt % nano-TiO_2_ showed uniform distribution with the least agglomeration was selected for EDX analysis. Thus, the SEM image shown in Figure 3B was selected for X-ray mapping analysis. The X-ray mapping in Figure 3C shows the presence of three main elements C, O, and Ti represented by dark purple, green, and dark teal colors with their respective K and M lines of the developed COC/nano-TiO_2_ film and their uniform distribution. The individual presence of C, O, and Ti elements and their uniform distribution in nanocomposite film are shown in Figure 3D–F. Thus, the mixing process of COC polymer and nano-TiO_2_ was adequate by the solution casting method, resulting in well dispersion of nano-TiO_2_ inside the COC polymer matrix.

The elemental compositional analysis was carried out for pure COC and COC/3 wt % nano-TiO_2_ film, and results are presented in Table 1. The main component of the COC polymer matrix is C with 97.1 wt % and agrees with nominal calculations as H cannot be detected by EDX which should be considered as the second main component. The concentration of O (2.9 wt %) was also detected in the pure COC matrix and might be due to contamination.^39^ The EDX spectrum was recorded for the developed nanocomposite film (3 wt % nano-TiO_2_), and the presence of C, O, and Ti can be seen in Figure 3G. Thus, EDX analysis revealed an increase in the concentration of O to 5.37 wt % which could be attributed to the addition of nano-TiO_2_ to COC polymer matrix. The concentration of C is slightly reduced and might be due to the increase in the concentration of O. Disregarding the COC matrix, and the compositional investigation showed that the ratio of average weight percent content of Ti to O is around 1:2, suggesting that the samples have good chemical stoichiometry.^40^

**Table 1:** Elemental Detection and Weight Percent Composition Using EDX Analysis

	elemental composition (wt %)		
sample	C	O	Ti
pure COC	97.1	2.9	0
COC/3 wt % nano-TiO_2_	91.70	5.37	2.93

**Figure 4:**
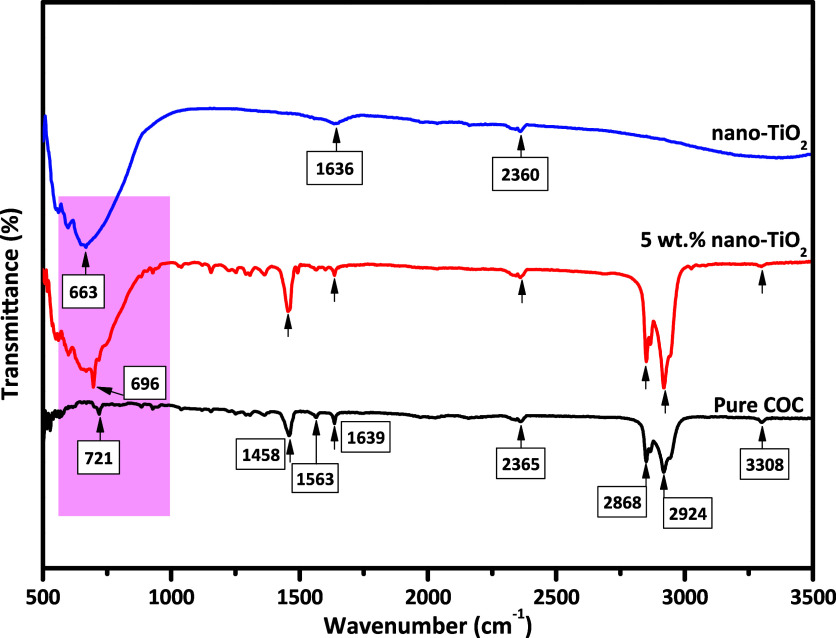
FTIR spectra of nano-TiO_2_, pure COC polymer, and COC/5 wt % nano-TiO_2_ nanocomposite film.

Figure 4 shows FTIR spectra of pure COC polymer, nano-TiO_2_, and COC/5 wt % nano-TiO_2_ nanocomposite films. The spectrum of nano-TiO_2_ is consistent, as reported in the literature.^38^ A band appearing at 663 cm^–1^ represents Ti–O bond confirmation that indicates the anatase structure of nano-TiO_2_.^41^ The absorption band at 1636 cm^–1^ is due to the absorption of water molecules during analysis from the atmosphere, and it confirms the existence of a hydroxyl group on the surface of nano-TiO_2_ (Ti–OH). The absorption band observed at 2360 cm^–1^ is likely caused by the distinctive frequency of organic species that remained, even after washing with ethanol and distilled water. This particular band is attributed to the stretching vibrations of alkane groups (C–H stretching vibrations).^32^ FTIR spectrum of the COC polymer shows absorption bands at 1458 and 1639 cm^–1^ that relate to C–H vibration of the methylene group and ethylene-norbornene conjugation, respectively.^21^ Moreover, the CH_2_ stretching mode-related vibrational band appears at 2924 cm^–1^, which is followed by the norbornene-related C=C absorption band appearing at 1563 cm^–1^.^21,26^

In FTIR spectra of nanocomposite film, the following phenomena can be observed: the peak in the pristine nano-TiO_2_ spectrum at 1636 cm^–1^ disappears, while the intensity of the peak at 663 cm^–1^ slightly decreases and there is also a slight shift in the peak position of the COC polymer from 721 to 695 cm^–1^, suggesting some level of interfacial interaction among the COC polymer and nano-TiO_2_ particles. FTIR spectrum of the COC/nano-TiO_2_ nanocomposite (5 wt % nano-TiO_2_) film exhibits no new absorption band formation, indicating that the addition of nano-TiO_2_ did not change the functional characteristics of the COC polymer. Therefore, no change in the chemical structure of any component of the nanocomposite film is observed. These findings are in accordance with the results obtained by Alrahlah et al.,^42^ where PMMA/TiO_2_ nanocomposite films were prepared where FTIR spectra of nanocomposite films reported no new absorption band formation.

### 3.2 Water Vapor Permeability and Oxygen Permeability

The variations in WVP and OP values versus the concentration of nano-TiO_2_ in COC polymer-based nanocomposite films are shown in Table 2. The results indicate that due to the utterly amorphous nature of COC polymer, it has higher WVP and OP values than nanocomposite films.^26^ The loadings of nano-TiO_2_ in COC polymer up to 5 wt % decreased WVP values from 80.67 ± 0.2 to 29.99 ± 0.6 (g/m^2^ h) and OP values from 7.66 ± 0.4 to 2.78 ± 0.4 (mg/mL), respectively. Zhou et al.^43^ reported similar results, where WVP values were decreased gradually by increasing the concentration of TiO_2_ nanoparticles in kefiran–whey protein isolate (WPI) film. The reduction in WVP and OP values was attributed to the addition of various concentrations of nano-TiO_2_ that caused blockage of micro paths in nanocomposite films. This led to hindered diffusion of water and oxygen molecules because now they had to follow a tortuous path through the COC polymer.^33,37^

**Table 2:** WVP and OP Values of COC/Nano-TiO_2_ Nanocomposite Films

sr. no	samples	WVP (g/m^2^ h)	OP (mg/mL)
1	pure COC	80.67 ± 0.2	7.66 ± 0.4
2	1 wt % nano-TiO_2_	54.55 ± 0.3	4.6 ± 0.2
3	3 wt % nano-TiO_2_	41.10 ± 0.7	3.7 ± 0.2
4	5 wt % nano-TiO_2_	29.99 ± 0.6	2.78 ± 0.4

### 3.3 Optical Transmittance

**Figure 5:**
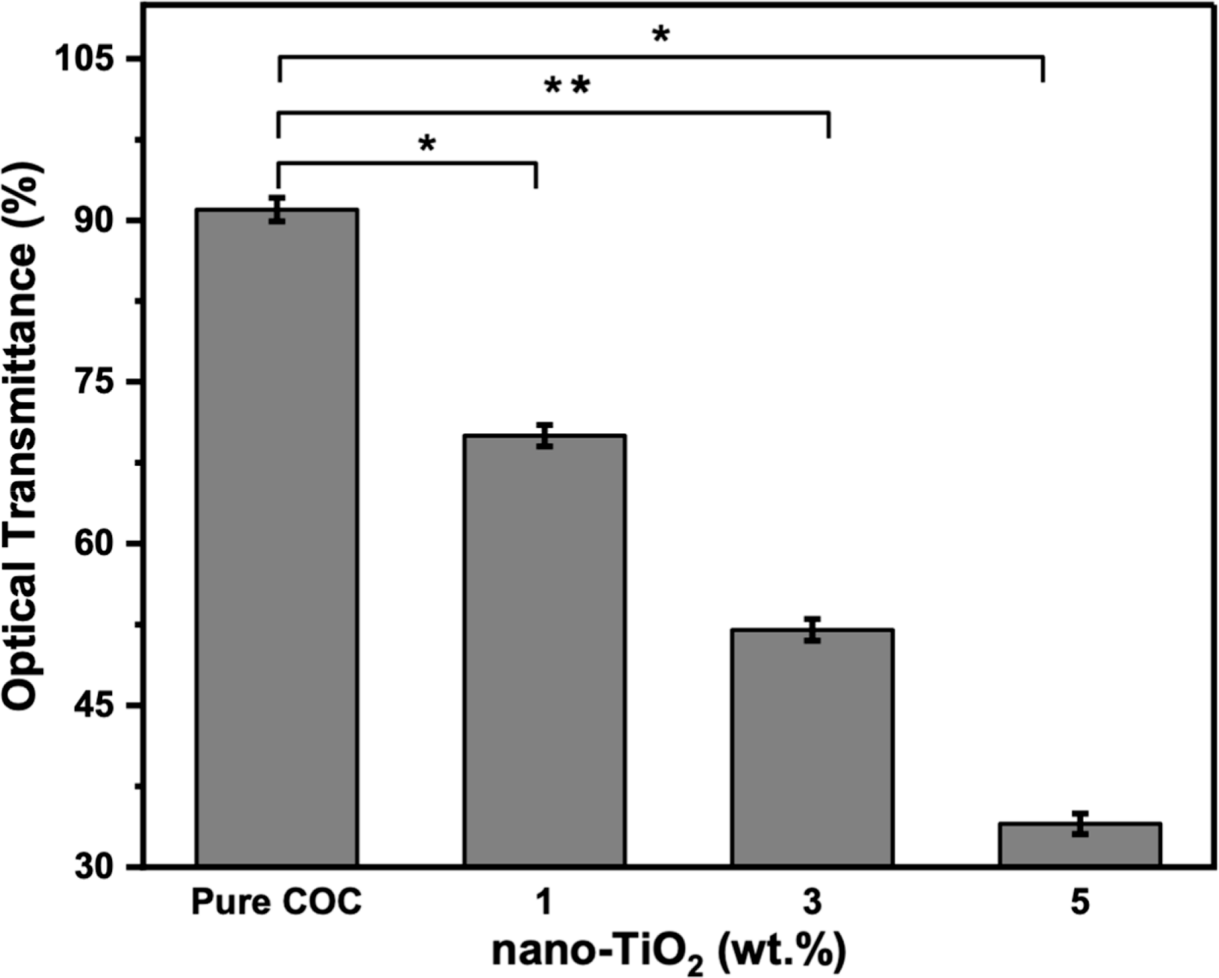
Optical transmittance (%) of COC/TiO_2_ nanocomposite at different nano-TiO_2_ concentrations with error bars representing ± SD. Data are statistically significant (*p < 0.05, **p < 0.01, and ***p < 0.001).

The optical transmittance values of pure COC and nanocomposite films were determined in the visible range (400–800 nm) from their respective spectra at five selected wavelengths (400, 500, 600, 700, and 800 nm). The average transmittance values of these specific wavelengths are presented in Figure 5. The pure COC film exhibited high optical transparency, approximately 91%, as there were no light-blocking particles present. When a low concentration of nano-TiO_2_ (1 wt %) was added to the COC film, there was a decrease in optical transparency (70% approx.), but at higher concentrations, the optical transparency decreased drastically, with a minimum transmission value of approximately 34% for films containing 5 wt % nano-TiO_2_ content. The literature reports that the optical transmittance of nanocomposite films is influenced by various factors, including the properties of the materials used, such as the particle size and refractive index of the components, and the fabrication process, such as the thickness of the nanocomposite, filler concentration, surface roughness, and nanoparticle dispersion.^44^ The decrease in the transmittance in the nanocomposite film is mainly due to light scattering caused by randomly dispersed spherical nano-TiO_2_ particles. However, the scattering losses are less at low concentrations due to the uniform dispersion of nano-TiO_2_ in the COC polymer matrix. However, at higher nano-TiO_2_ concentrations, the nanoparticles are more likely to aggregate, resulting in the formation of larger light scattering centers that cause more optical scattering, which leads to greater transmission loss and opacity in the nanocomposite film.^9^ Another important factor is that the large difference in refractive indices of nano-TiO_2_ (2.5) and COC polymer (1.53) results in strong optical scattering, reducing the transparency of the nanocomposite films.^45^

### 3.4 Mechanical Properties

**Figure 6:**
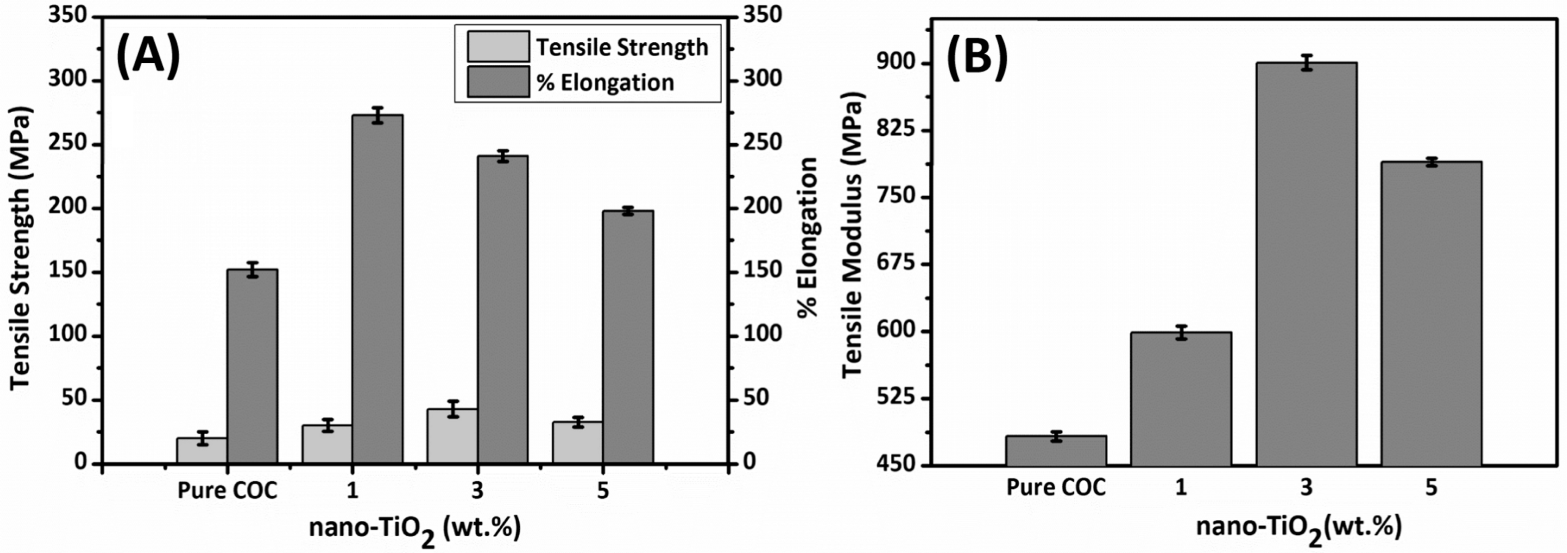
Mechanical studies showing (A) TS and % elongation and (B) tensile modulus of the COC polymer and COC/nano-TiO_2_ nanocomposite films.

Mechanical studies comprising TS, tensile modulus, and % elongation of COC/nano-TiO_2_ nanocomposite films having varying concentrations (1–5 wt %) of nano-TiO_2_ are presented in Figure 6. Results indicate that the overall tensile properties of the COC polymer film improved by the addition of nano-TiO_2_. For instance, the TS of COC polymer increased by 113% from 12.1 to 42.86 MPa upon adding 3 wt % nano-TiO_2_. With the further addition of nano-TiO_2_ (5 wt %), it decreased to 32.59 MPa, still 63.1% higher than COC polymer as shown in Figure 6A. A similar trend is true for tensile modulus, which increased by 81.3% from 483 to 901 MPa, respectively, upon the addition of nano-TiO_2_ up to 3 wt % and decreased to 790 MPa upon further addition of nano-TiO_2_ (5 wt %) but still 63.5% higher than the COC polymer (Figure 6B). However, in the case of % elongation at break, it increased from 152% to 241% upon the addition of 3 wt % nano-TiO_2_ and then decreased to 198% at 5 wt % loading (Figure 6A). These findings make the COC/nano-TiO_2_ nanocomposite system strong, stiff, tough, and ductile, which could be attributed to both strengthening and toughening mechanisms in the nanocomposite system. Generally, it has been seen that mostly nanocomposite systems show an increase in stiffness and a decrease in toughness properties; however, few studies reported an increase in stiffness and toughness behavior.^46,47^ The strengthening mechanism based on the reinforcement of TS and tensile modulus of nanocomposite films is directly linked with interfacial interaction among nano-TiO_2_ and the COC polymer matrix. At lower nano-TiO_2_ concentrations ranging from 1 to 3 wt %, nano-TiO_2_ particles are uniformly dispersed within the COC polymer matrix instead of agglomerating with each other, thus acting as a reinforcement agent, strengthening the polymer film network.

Moreover, at lower concentration of nano-TiO_2_, COC polymer chains are anchored near nano-TiO_2_, so load can be successfully transferred across the polymer/nano-TiO_2_ interface to nano-TiO_2_, leading toward improvements in TS and tensile modulus. Upon further addition of nano-TiO_2_ up to 5 wt %, the distance among nano-TiO_2_ decreases, which causes agglomeration as a result of which collision between nanoparticles starts.^1,48,49^ These agglomerates of nano-TiO_2_ act as stress concentration centers and cause nonuniform stress distribution within the nanocomposite structure. As a result, the resistance of the nanocomposite film network decreases against fracture, which leads to a decrease in TS and tensile modulus. These results are in accordance with the studies conducted by Zhou et al.,^35^ where they found that TS of WPI was increased upon addition of low concentration of TiO_2_ nanoparticles and decreased at higher concentration due to the formation of agglomerations. Kasgoz et al.^22^ fabricated Topas/carbon black (CB) composite films in which Young’s modulus was increased by 40% upon adding 30 wt % filler concentration. Similarly, Siripatrawan and Kaewklin^9^ explained the mechanical properties analysis of Chitosan-TiO_2_ nanocomposite films, where TS reached a maximum value of 16.43 MPa upon 1 wt % nano-TiO_2_ loading and then decreased upon 2 wt % loading. The toughening mechanism shown by COC/nano-TiO_2_ nanocomposite films is due to delay in crack propagation, possibly because of energy dissipation through the successful transmission of load across COC/nano-TiO_2_ interface as it avoids the initiation of stress concentration sites, thus making system tough along with stiffness.^23,24,26^

### 3.5 Antibacterial Studies

**Figure 7:**
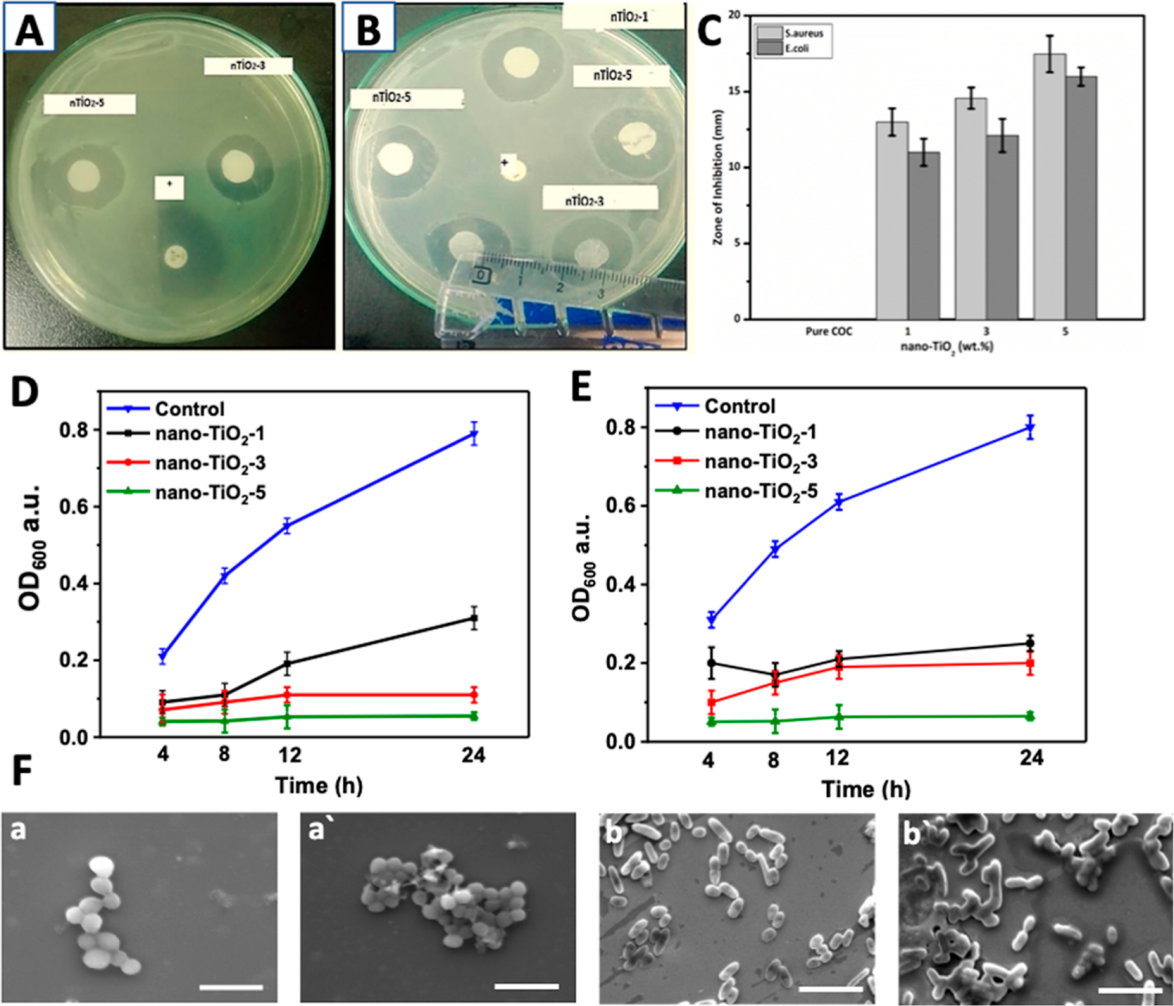
Antibacterial studies. Zone of inhibitions formed by COC-nano-TiO_2_ nanocomposite films with varying contents of TiO_2_ NPs against (A) *E. coli* and (B) *S. aureus*. (C) Quantitative measurements of inhibition zones. Antibacterial activity of nanocomposite films measured at different intervals against (D) *E. coli* and (E) *S. aureus*. While (F) represents SEM images of bacterial cells before and after treatment with nanocomposites (scale bar: 5 μm).

The antibacterial properties of nanocomposite films were studied by the well diffusion method. The developed nanocomposite films with 1, 3, and 5 wt % nano-TiO_2_ in the COC polymer matrix are represented by nTiO_2_-1, nTiO_2_-3, and nTiO_2_-5 labels in Figure 7A,B. COC/nano-TiO_2_ nanocomposite films at all concentrations of nano-TiO_2_ exhibit a zone of inhibition against both strains of bacteria used in this experiment (Figure 7A,B). Figure 7C shows that the antibacterial activity of the nanocomposite film is directly linked with nano-TiO_2_ concentration. The diameter of zone of inhibition increased with increase in concentration of nano-TiO_2_ in the COC polymer film from 1 to 5 wt %. To further investigate the antibacterial properties of nanocomposite films, 1 mL of bacterial solution is added to 24-well plates with nanocomposite films. Afterward, these plates were placed on the mechanical shaker with the speed of about 90 rpm for various time intervals of 4, 8, 12, and 24 h at 37 °C temperature. Later on, OD values at 600 nm were determined to evaluate the antibacterial activity of these nanocomposite films. The results obtained from this experiment are displayed in Figure 7D,E and are in complete agreement with the findings obtained from the above explained well diffusion method.

SEM analysis was carried out for further deep investigations to have better understanding of how these nanocomposite films interact with bacterial cells and cause their death (Figure 7F). It has been observed that healthy cells before interaction with nanocomposite film shows regular surface morphology without any physical damage (Figure 7Fa,b). However, bacterial cells after interaction with 5 wt % TiO_2_ nanocomposite film for 5 h show visible changes in their morphologies (Figure 7Fa′,b′). The cell wall/membrane of most of the cells was broken with leakage of cytoplasm and cellular components everywhere. The bacterial cells were out of shape, and they lose their regular cell morphology and were completely disintegrated. The antibacterial activity of these nanocomposite films is mainly attributed to the existence of nano-TiO_2_, which is well-known for its antibacterial activity. Several studies have reported the mechanisms of antibacterial activity of photocatalytic titanium dioxide nanoparticles that mainly involve interactions between the nanoparticles and biological molecules.^50^ Microbes have a negative charge, while metal oxide nanoparticles have a positive charge, leading to an electromagnetic reaction between the two upon contact with the treated material surface. This reaction oxidizes the microbes and ultimately results in cell death.

The antibacterial activity of nanocomposite films depends upon nano-TiO_2_ that generates reactive oxygen species (ROS). These ROS including hydroxyl (OH^•^) and hydrogen peroxide (OH_2_^•–^) radicals have high oxygen potential that is detrimental to the DNA of microorganisms.^13^ Thus, these ROS are responsible for the oxidative damage of the organic structure of microorganisms and eventually cell death.^51^ Additionally, the TiO_2_ nanoparticles can also interact with phosphorus- or sulfur-containing compounds such as DNA and proteins, inhibiting DNA replication and inactivating proteins. These interactions can also cause pits in bacterial cell walls, increasing cell permeability and eventually leading to cell death.^52^ Thus, the electromagnetic attraction between microorganisms and metal oxide nanoparticles produces a synergistic effect that may result in the deactivation of cells at both the regulatory network and signaling levels. This, in turn, may reduce the functioning of the respiratory chain and hinder the absorption and transportation of iron and phosphorus.^53^ These changes in the cellular processes, along with significant modifications to the cell wall and membrane, are the key reasons for the biocidal properties of TiO_2_ nanoparticles.

### 3.6 Cytotoxicity Studies

**Figure 8:**
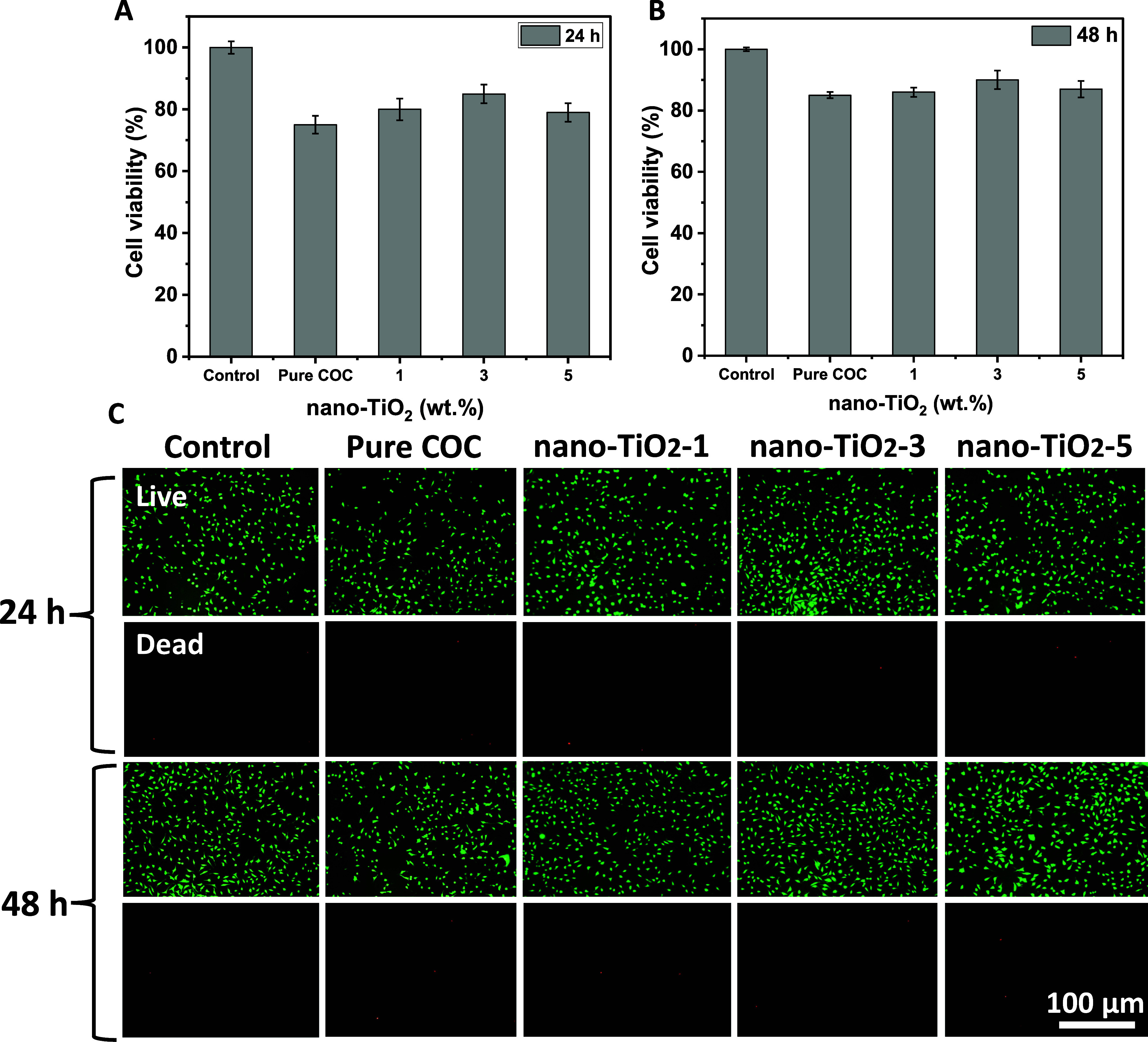
Cell viability analysis of pure COC and nanocomposite with varying contents of nano-TiO_2_ films when incubated with L929 cells for (A) 24 h and (B) 48 h. While (C) fluorescent microscopic images showing live and dead L929 cells when cultured for 24 and 48 h incubation period.

Although the nanocomposite system displayed remarkable antibacterial properties, it is crucial to highlight its biocompatibility to use this system as a biomaterial for various biomedical applications. Cytotoxicity studies of pure COC film and COC film with varying contents of nano-TiO_2_ particles were carried out at two time periods (24 h) and (48 h) by using an L929 cell line through MTT assay, whereas pure polystyrene (PS) was used as a positive control. The results presented in Figure 8A demonstrate that pure COC film, as well as all nanocomposite films with varying concentrations of TiO_2_ nanoparticles, exhibit cell viabilities of more than 75% indicating the noncytotoxic nature of these nanocomposite systems. It has been observed that with an increase in incubation time, cell viabilities increased, as after 48 h, the cell viabilities increased more than 80% (Figure 8B). The live/dead fluorescent staining assay images are shown in Figure 8C, where dead cells were stained with red fluorescent and live cells were stained with green fluorescent. The images show that live cells proliferate, grow, and adhere as well as spread well on surface of nanocomposite films. The biocompatible nature of COC polymer has been reported in the literature.^26^ The cytotoxicity studies of nano-TiO_2_ have been extensively studied, and they are found to be noncytotoxic and did not cause any oxidative stress or histopathological changes in liver, heart, or kidney of mammalians.^54^ Our experimental findings are in agreement with previous reports. It has been observed that cell viability of nanocomposite films was enhanced with nano-TiO_2_ incorporation. This might be due to increase in surface roughness as we increased the TiO_2_ nanoparticle concentration, which leads to more cell attachment. Moreover, as discussed previously, nano-TiO_2_ acts as a reinforcing agent that leads to an enhancement in the mechanical strength of the nanocomposite system, which in turn helps enhance the film–cell interaction. However, in the case of nanocomposite film with 5 wt % nano-TiO_2_, its cell viability was slightly less than the nanocomposite system with 3 wt % TiO_2_. This might be due to the blockage of cell adhesion sites due to excessive concentration of TiO_2_ nanoparticles.^55−57^

## 4 Conclusions

The incorporation of TiO_2_ nanoparticles into the COC matrix successfully enhanced the multifunctional properties of the nanocomposite films. At an optimal concentration of 3 wt %, the films significantly improved barrier performance, reducing water vapor and OP by 62.8% and 63.7%, respectively. Mechanical properties were also markedly enhanced, with TS and modulus increasing by 113% and 81.3%, respectively. Furthermore, the nanocomposites exhibited broad-spectrum antibacterial activity against *E. coli* and *S. aureus* while maintaining excellent biocompatibility, as confirmed by cytotoxicity studies. These results highlight the potential of COC/TiO_2_ nanocomposites for advanced applications in biomedical fields, such as antibacterial coatings and wound healing patches, as well as in high-performance packaging materials. This study provides a promising foundation for future research on nanocomposite optimization and in vivo evaluation to explore their practical applications further.
